# Repeatability of apparent diffusion coefficient and intravoxel incoherent motion parameters at 3.0 Tesla in orbital lesions

**DOI:** 10.1007/s00330-017-4933-6

**Published:** 2017-07-04

**Authors:** Augustin Lecler, Julien Savatovsky, Daniel Balvay, Mathieu Zmuda, Jean-Claude Sadik, Olivier Galatoire, Frédérique Charbonneau, Olivier Bergès, Hervé Picard, Laure Fournier

**Affiliations:** 1 Department of Radiology, Fondation Ophtalmologique Adolphe de Rothschild, 29 rue Manin, 75019 Paris, France; 2 Université Paris Descartes Sorbonne Paris Cité, INSERM UMR-S970, Cardiovascular Research Centre – PARCC, Paris, France; 3 Department of Orbitopalpebral Surgery, Fondation Ophtalmologique Adolphe de Rothschild, Paris, France; 4 Clinical Research Unit, Fondation Ophtalmologique Adolphe de Rothschild, Paris, France; 5 Assistance Publique-Hôpitaux de Paris, Hôpital Européen Georges Pompidou, Radiology Department, Université Paris Descartes Sorbonne Paris Cité, Paris, France

**Keywords:** IVIM, MRI, DWI, Orbit, Feasibility

## Abstract

**Objectives:**

To evaluate repeatability of intravoxel incoherent motion (IVIM) diffusion-weighted imaging (DWI) parameters in the orbit.

**Methods:**

From December 2015 to March 2016, 22 patients were scanned twice using an IVIM sequence with 15b values (0–2,000 s/mm^2^) at 3.0T. Two readers independently delineated regions of interest in an orbital mass and in different intra-orbital and extra-orbital structures. Short-term test-retest repeatability and inter-observer agreement were assessed using the intra-class correlation coefficient (ICC), the coefficient of variation (CV) and Bland-Altman limits of agreements (BA-LA).

**Results:**

Test-retest repeatability of IVIM parameters in the orbital mass was satisfactory for ADC and D (mean CV 12% and 14%, ICC 95% and 93%), poor for *f* and D*(means CV 43% and 110%, ICC 90% and 65%). Inter-observer repeatability agreement was almost perfect in the orbital mass for all the IVIM parameters (ICC = 95%, 93%, 94% and 90% for ADC, D, *f* and D*, respectively).

**Conclusions:**

IVIM appeared to be a robust tool to measure D in orbital lesions with good repeatability, but this approach showed a poor repeatability of *f* and D*.

***Key Points*:**

• *IVIM technique is feasible in the orbit*.

• *IVIM has a good*–*acceptable repeatability of D* (*CV range 12*–*25* %).

• *IVIM interobserver repeatability agreement is excellent* (*ICC range 90*–*95* %).

• f or D* *provide higher test*-*retest and interobserver variabilities*.

**Electronic supplementary material:**

The online version of this article (doi:10.1007/s00330-017-4933-6) contains supplementary material, which is available to authorized users.

## Introduction

Diffusion-weighted imaging (DWI) using magnetic resonance imaging (MRI) was described by Le Bihan in 1986 as being sensitive to displacement of water protons in tissues due to both random Brownian motion as well as capillary perfusion [1, 2]. Intravoxel incoherent motion (IVIM) imaging uses multiple b values with a biexponential model to quantify both phenomena. The DW signal is the result of a ‘pure’ diffusion fraction and a perfusion-dominated pseudo-diffusion fraction, yielding three parameters: the ‘pure’ diffusion coefficient (D), the perfusion fraction (*f*) and the pseudo-diffusion coefficient (D*) [1].

$$ S(b)= S(0)\left[ f\kern0.5em {e}^{- bD*}+\left(1- f\right){e}^{- bD}\right] $$

This model has been shown to provide a pertinent description of the DW signal measured in highly vascular organs [3]. Due to both improvement of MR devices and post-treatment softwares, IVIM has become more available in clinical practice. It has been tested and proved useful in a variety of organs, such as prostate, liver, abdomen, kidney and pelvis, and also in the head and neck [4–8], to diagnose liver diseases, characterise tumours, or assess and monitor tissue response to treatment [4–8]. However, one issue is repeatability, which has been evaluated over time in brain imaging [9, 10] or abdominal imaging [11–15].

In the orbit, only a few studies evaluated the DWI, showing its usefulness when visualising and diagnosing tumours [16, 17]. An ADC value <1 10^-3^ s/mm^2^ at b=1,000 s/mm^2^ were considered optimal thresholds to predict overall malignancy [17] and a low ADC <0.6 10^-3^ s/mm^2^ at b=1,000 s/mm^2^ was reported to have good accuracy in distinguishing benign from malignant orbital lymphoproliferative disorders [18]. Also, two recent studies combining morphological characteristics to DWI and dynamic contrast-enhanced perfusion showed that ADC alone yielded the optimal sensitivity in differentiating malignant from benign orbital lymphoproliferative disorders [19, 20]. However, IVIM has never been tested in the orbits, and it makes sense to test IVIM repeatability in orbital anatomy/disease in order to understand technical limitations before its use in future studies.

The aim of our study was to prospectively evaluate robustness of IVIM-derived parameters in the orbit by evaluating the short-term test-retest and inter-observer repeatability of IVIM parameters and apparent diffusion coefficient (ADC) of orbital lesions and normal intra-orbital and extra-orbital structures at 3.0T.

## Materials and methods

### Study design

We conducted a prospective study in a tertiary referral centre specialising in ophthalmic diseases (NCT02401906). This study was prospectively approved by our institutional Research Ethics Board and adhered to the tenants of the Declaration of Helsinki (IRB 2015-A00364-45). Signed informed consent was obtained from all subjects.

From December 2015 through March 2016, 22 patients were included in the study. Inclusion criteria were: (a) age over 18 years; (b) presence of an orbital mass. Patients with an MR contraindication such as implanted pacemakers, the presence of other metallic foreign bodies or claustrophobia were excluded.

### MR imaging

All MRIs were performed on the same 3 Tesla Philips INGENIA device with a 32-channel head coil (Philips Medical Systems, Best, The Netherlands). All patients had the same MRI protocol including two IVIM acquisitions acquired with 15 b values, ranging from 0 to 2,000 s/mm^2^. The b values distribution was chosen to cover both the initial pseudodiffusion decay (≤200 s/mm^2^) and the molecular diffusion decay (>200 s/mm^2^). We used a large number of lower b values for more accurate calculation of IVIM parameters. The first acquisition was acquired at the beginning of the examination and the second one immediately before contrast injection, with a median delay between the two scanning procedures of 17 min, providing two distinct data sets of IVIM. Patients were asked to look at a fixed point during the acquisitions in order to prevent kinetic artefacts generated from eye movements. They were also asked to move between the two sequences and were repositioned for the second IVIM acquisition. Technical specifications of the MRI protocol are provided in Table 1.

**Table 1:** Specifications of the intravoxel incoherent motion (IVIM) sequence parameters

IVIM	
TR/TE (ms)	2,639 / 74
Section thickness (mm)	3
Number of slices	210
Gap (mm)	1
Bandwith (Herz)	1,695
Number of excitations	1
Number of b-values	15
b values (s/mm^2^ )	0, 10, 20, 40, 80, 110, 140, 170, 200, 300, 400, 600, 800, 1,000, 2,000
Field of view (mm^2^)	122 × 122
Matrix	176 × 122
Parallel acceleration factor	SENSE = 2
Acquisition duration (s)	143

### Image analysis

Two radiologists, blinded to patient ID, medical history and laboratory results, read independently and in random order the IVIM sequences. The first senior neuroradiologist was specialized in orbital imaging with 7 years of experience (AL), and the second was a senior radiologist with no experience in orbital imaging (LF).

**Fig. 1:**
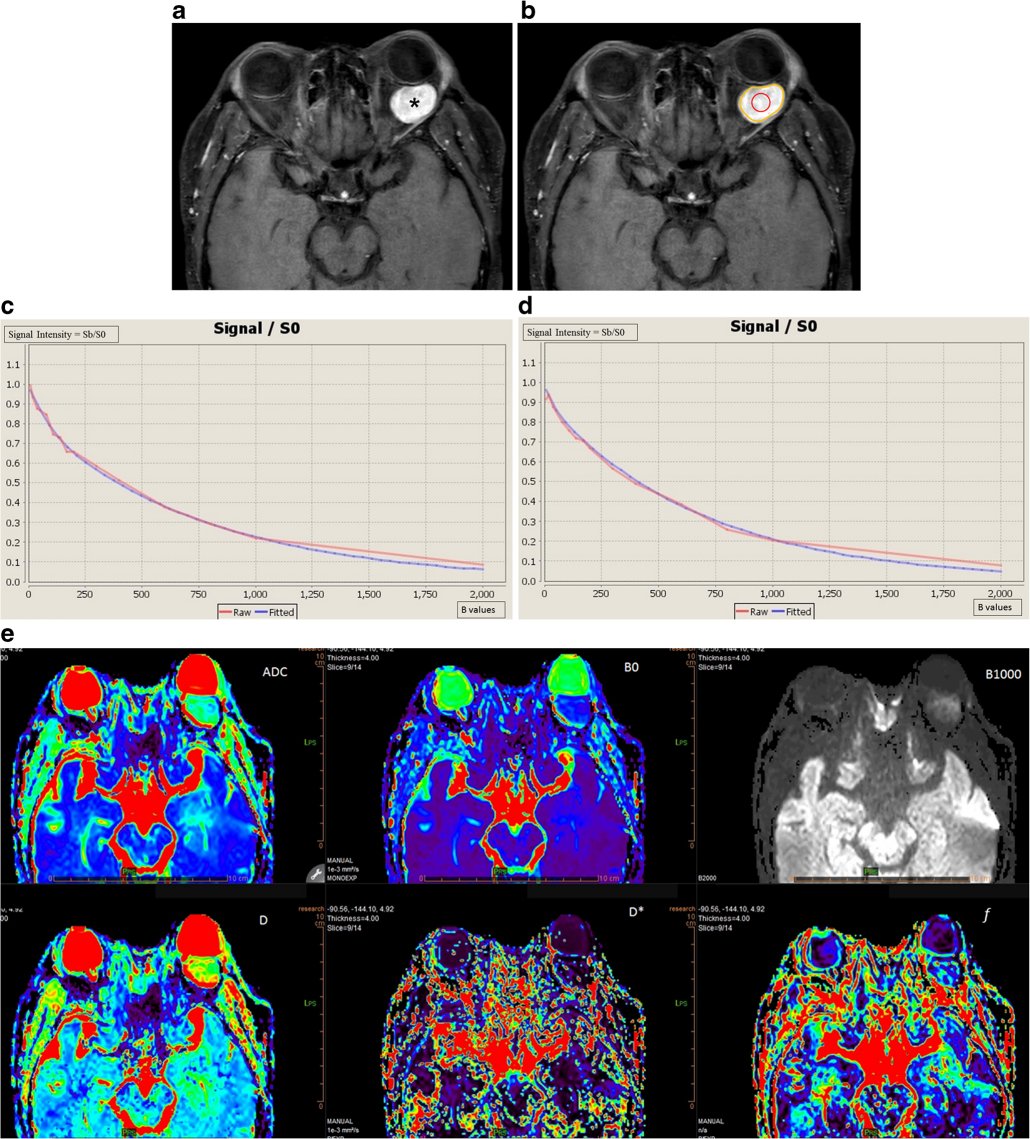
A 47-year-old man with a cavernous haemangioma in his left orbit (asterisk), displacing the eye anteriorly, as shown on the axial post-contrast T1 WI (**a**). Placement of a freehand large region of interest (ROI) (yellow line) and of a circular small ROI (red circle) inside the mass are shown (**b**). Test (**c**) and retest (**d**) bi-exponential fitting curves using b values are shown. Coloured intravoxel incoherent motion (IVIM) parametric map is displayed (**e**)

All the post-processing steps were performed using the Olea Sphere® software (v3.0, Olea Medical, La Ciotat, France), implementing a Bayesian probability-based algorithm using all 15 b-values to fit a biexponential diffusion model to each voxel within a region of interest (ROI) for the calculation of IVIM parameters (D, D* and *f*), and a monoexponential diffusion model for the calculation of ADC (Fig. 1).

The operators independently drew seven ROIs on each imaging set: two ROIs inside the orbital mass, the first one encompassing the maximum area of the orbital mass (ROI 1) and the second one a circular ROI at the centre of the orbital mass without consideration for the zones with restricted diffusion or with enhancing nodules if present (ROI 2) (Fig. 1), three ROIs in non-tumoral orbital structures: in the contralateral lacrymal gland (ROI 3), in the contralateral medial (ROI 4) and lateral (ROI 5) extra-ocular muscles, and two ROIs outside the orbit: one ROI in the temporal muscle (ROI 6) and one ROI in the contralateral temporal lobe white matter (ROI 7). The five last ROIs were drawn in order to analyse their repeatability, in case they might be used in further studies as structures of reference to normalise IVIM values (as is done sometimes in brain studies [10, 21]). Size and location of the ROIs in the orbital and encephalic locations was kept constant across patients to every possible extent, except for ROI 1, which encompassed the largest possible portion of the mass on the slice including its largest diameter. ROIs were placed on the transverse b0 image and propagated to all b-values, ADC and IVIM parametric maps. Readers were free to review the other structural images to ease the drawing of the ROI. A direct registration between the structural and the IVIM images was not possible due to the distortions seen on the IVIM images.

In addition, given that parallel imaging was used for DWI, we used the difference method to estimate the signal to noise ratio (eSNR) in the orbital mass [22].

### Statistical analysis

Data were analysed using the R software package (R Foundation for Statistical Computing, Vienna, Austria). Mean IVIM values and CVs were computed for each reader individually, then averaged for both readers. Test-retest repeatability of IVIM parameters was assessed by calculating the coefficient of variation (CV, computed as standard deviation (SD) divided by the mean), the intra-class correlation coefficient (ICC), and the 95% Bland-Altman limits of agreements (BA-LA) [23, 24] for each reader individually, then averaged for both readers, as per the recommendations of the Quantitative Imaging Biomarkers Alliance (QIBA) [25]. The parameters’ repeatability was defined as excellent when CV was ≤10%, good when CV was between 10–20%, acceptable when CV was between 20–30%, and poor when CV was >30% [24]. The inter-observer repeatability agreement was assessed by calculating the ICC and was interpreted as follows: 0.0–0.2:poor correlation; 0.21–0.4:fair correlation ;0.41–0.6:moderate correlation ; 0.61–0.8:good correlation ;0.81–1:almost perfect correlation [23].

## Results

Twenty-two consecutive patients were included in the study (nine males and 13 females, median age 51 years, range 41–62). Six patients had a histologically-proven orbital lymphoma, six patients had an orbital inflammation, four patients had a cavernous haemangioma, two had a dacryoadenitis, two had an orbital metastasis, one had a muscular granuloma and one had an orbital sarcoma. The quality of ADC and IVIM parametric maps was considered good for subsequent analysis for all patients, and no patient had to be secondarily excluded from the study because of artefacts masking or distorting orbital tumours.

Mean (SD) ROI size was 86 (36) pixels for ROI 1, 23.5 (9) for ROI 2, 21 (9) for ROI 3, 27 (10) for ROI 4, 22.5 (8) for ROI 5, 127.5 (48) for ROI 6 and 601 (114) for ROI 7.

Mean (SD) IVIM parameter values inside all orbital lesions were calculated as follows: 1.26 (0.35), 1.02 (0.27), 0.18 (0.07) and 14.6 (7.9) for ADC, D, *f* and D*, respectively. The mean IVIM parameters values and SDs inside the other ROIs are shown in Table 2. The average eSNR inside the orbital mass at b=2,000 s/mm^2^ was 56.6 (SD 3.5)

**Table 2:** Mean intravoxel incoherent motion (IVIM) parameter values (of all measurements)

Mean values				
Anatomical region	ADC (10^-3^ s/mm^2^) (SD)	D (10^-3^ s/mm^2^) (SD)	*f* (%) (SD)	D*(10^-3^ s/mm^2^) (SD)
ROI 1. Freehand large ROI inside the mass	1.26 (0.35)	1.02 (0.27)	0.18 (0.07)	14.6 (7.9)
ROI 2. Circular small ROI inside the mass	1.25 (0.38)	1.02 (0.3)	0.17 (0.08)	14.2 (9.4)
ROI 3. Lacrymal gland	1.5 (0.68)	1.13 (0.28)	0.2 (0.08)	15.4 (7.8)
ROI 4. Medial extra-ocular muscle	1.74 (0.57)	1.27 (0.21)	0.21 (0.08)	11.6 (5.1)
ROI 5. Lateral extra-ocular muscle	1.54 (0.27)	1.17 (0.21)	0.2 (0.06)	9.8 (4.9)
ROI 6. Temporal muscle	1.63 (0.29)	1.16 (0.13)	0.23 (0.07)	14.6 (5.6)
ROI 7. Temporal lobe	0.89 (0.07)	0.75 (0.04)	0.13 (0.05)	20.2 (6.7)

*ADC* apparent diffusion coefficient, *D* ‘true’ diffusion coefficient, D* pseudodiffusion coefficient, *f* perfusion fraction, *SD* standard deviation, *ROI* region of interest

### Test-retest repeatability

**Fig. 2:**
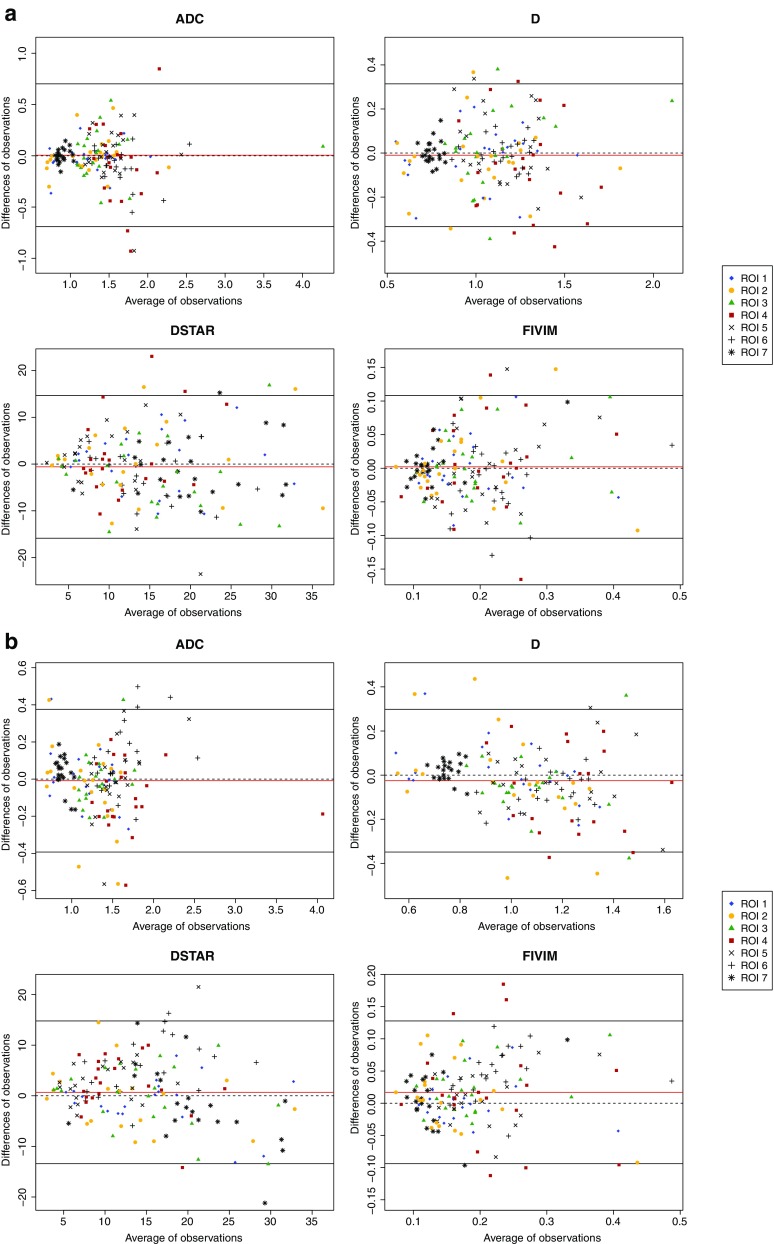
Bland-Altman plots showing test-retest repeatability (**a**) and inter-observer repeatability agreement (**b**) of apparent diffusion coefficient (ADC), ‘true’ diffusion coefficient (*D*), pseudodiffusion coefficient (D*), perfusion fraction (*f*), for all regions of interest (ROIs)

Repeatability of IVIM parameters in the orbital mass was good for ADC and D (mean CV 12% and 14%, ICC 95% and 93%, BA-LA : [−0.35; 0.35] and [−0.36; 0.27]) and poor for *f* and D*(mean CV 43% and 110%, ICC 90% and 65%, BA-LA : [−0.10; 0.10] and [−23.1; 20.9]) (Fig. 2.a). There was a significant correlation between ADC and D values in the orbital mass (p<0.001).

Regarding the structures which could be used as references, repeatability was good in the lacrymal gland for ADC and D (mean CV 18% and 19%, ICC 94% and 86%, BA-LA : [−0.61; 0.75] and [−0.36; 0.45]), poor for *f* and D*(mean CV 51% and 130%, ICC 91% and 75%, BA-LA : [−0.09; 0.10] and [−17.6; 12.0]). Repeatability was acceptable in the extra-ocular muscles and the extra-orbital structures for ADC and D and poor for *f* and D*, with a slightly better repeatability for the lateral extra-ocular muscle (Supplementary Fig. 1). All detailed values are shown in Table 3.

**Table 3:** Test-retest intraclass correlation coefficients (ICCs), Bland-Altman limits of agreements (BA-Las), Bias and coefficients of variation (CVs) of intravoxel incoherent motion (IVIM)–diffusion-weighted imaging (DWI) values for each anatomical region

		ROI 1	ROI 2	ROI 3	ROI 4	ROI 5	ROI 6	ROI 7	Mean of all ROI
ICC (mean) [95% CI]	ADC	0.96 [0.91–0.98]	0.95 [0.87–0.98]	0.94 [0.85–0.97]	0.54 [0–0.81]	0.75 [0.39–0.90]	0.89 [0.70–0.96]	0.77 [0.45–0.91]	0.87 [0.82–0.90]
	D	0.94 [0.86–0.98]	0.93 [0.83–0.97]	0.86 [0.67–0.94]	0.72 [0.35–0.88]	0.71 [0.28–0.88]	0.94 [0.85–0.97]	0.59 [0; 0.83]	0.90 [0.87–0.93]
	PF	0.91 [0.78–0.96]	0.90 [0.77–0.96]	0.91 [0.79–0.96]	0.75 [0.40–0.90]	0.76 [0.41–0.90]	0.86 [0.64–0.94]	0.90 [0.77–0.96]	0.87 [0.83–0.91]
	D*	0.84 [0.62–0.93]	0.65 [0.15–0.86]	0.75 [0.41–0.90]	0.35 [0; 0.73]	0.32 [0–0.72]	0.76 [0.43–0.90]	0.78 [0.46–0.91]	0.73 [0.63–0.80]
BA-LA	ADC	[-0.29–0.26]	[-0.35–0.35]	[-0.61–0.75]	[-1.48–1.60]	[-0.52–0.55]	[-0.44–0.26]	[-0.13–0.13]	[-0.69–0.70]
	D	[-0.28–0.25]	[-0.36–0.27]	[-0.36–0.45]	[-0.48–0.35]	[-0.45–0.45]	[-0.12–0.13]	[-0.10–0.11]	[-0.33–0.31]
	PF	[-0.08–0.08]	[-0.10–0.10]	[-0.09–0.10]	[-0.15–0.17]	[-0.12–0.13]	[-0.12–0.08]	[-0.06–0.07]	[-15.9–14.7]
	D*	[-11.8–13.2]	[-23.1–20.9]	[-17.6–12.0]	[-14.7–17.7]	[-15.6–16.3]	[-12.3–8.0]	[-13.2–11.9]	[-0.10–0.11]
Bias (mean) [95% CI]	ADC	-0.01 [-0.08–0.05]	0.00 [-0.08–0.08]	0.07 [-0.09–0.22]	0.06 [-0.28–0.41]	0.01 [-0.11–0.14]	-0.09 [-0.17–-0.01]	0 [-0.03–0.03]	0.01 [-0.05–0.06]
	D	-0.02 [-0.08–0.04]	-0.04 [-0.11–0.03]	0.05 [-0.04–0.14]	-0.06 [-0.16–0.03]	0 [-0.10–0.10]	0 [-0.03–0.03]	0 [-0.02–0.03]	-0.01 [-0.04–0.02]
	PF	0 [-0.02–0.02]	0.00 [-0.02–0.03]	0.00 [-0.02–0.03]	0.01 [-0.02–0.05]	0.01 [-0.02–0.03]	-0.02 [-0.04–0.00]	0.01 [-0.01–0.02]	0 [-0.01–0.01]
	D*	0.71 [-2.13–3.54]	-1.1 [-6.08–3.89]	-2.78 [-6.14–0.57]	1.50 [-2.17–5.16]	0.35 [-3.26–3.96]	-2.15 [-4.45–0.15]	-0.64 [-3.48–2.19]	-0.59 [-1.83–0.65]
CV (mean) (SD)	ADC	0.21 (0.06)	0.12 (0.05)	0.18 (0.06)	0.22 (0.06)	0.22 (0.07)	0.33 (0.13)	0.23 (0.06)	0.22 (0.09)
	D	0.23 (0.06)	0.14 (0.05)	0.19 (0.06)	0.25 (0.07)	0.25 (0.06)	0.29 (0.06)	0.22 (0.05)	0.22 (0.07)
	PF	0.62 (0.18)	0.43 (0.17)	0.51 (0.15)	0.51 (0.13)	0.54 (0.1)	0.66 (0.11)	0.75 (0.14)	0.57 (0.17)
	D*	1.5 (0.4)	1.1 (0.4)	1.3 (0.4)	1.3 (0.4)	1.3 (0.4)	1.6 (0.2)	1.2 (0.2)	1.3 (0.4)

*CI* confidence interval, *ADC* apparent diffusion coefficient, *D* ‘true’ diffusion coefficient, D* pseudodiffusion coefficient, *f* perfusion fraction, *ROI* region of interest

### Inter-observer repeatability

Inter-observer repeatability was almost perfect in the orbital mass (ICC = 95%, 93%, 94% and 90% and BA-LA = [−0.30; 0.29], [−0.26 0.27], [−0.06; 0.07] and [−10.5; 8.6] for ADC, D, *f* and D*, respectively) (Fig. 2.b). It was almost perfect in the lacrymal gland for all the IVIM parameters (ICC = 80%, 84%, 90% and 84% and BA-LA = [−0.56; 0.44], [−32; 0.23], [−0.06; 0.10] and [−12.3; 12.1] for ADC, D, *f* and D*, respectively). It was almost perfect in the extra-ocular muscles and the temporal muscle for ADC, almost perfect in the lateral extra-ocular muscle and the temporal muscle for D and in the temporal muscle for *f*. This level of inter-observer repeatability was only seen for the extra-ocular muscles and the temporal muscle for D* (Supplementary Fig. 2). All detailed values are shown in Table 4.

**Table 4:** Interobserver repeatability agreement intraclass correlation coefficients (ICCs), Bland-Altman limits of agreements (BA-Las), Bias and coefficients of variation (CVs) of intravoxel incoherent motion (IVIM)–diffusion-weighted imaging (DWI) values for each anatomical region

		ROI 1	ROI 2	ROI 3	ROI 4	ROI 5	ROI 6	ROI 7	Mean of all ROI
ICC (mean) [95% CI]	ADC	0.95 [0.86–0.98]	0.88 [0.70–0.95]	0.80 [0.52–0.92]	0.97 [0.91–0.99]	0.88 [0.70–0.95]	0.88 [0.62–0.96]	0.57 [0–0.82]	0.95 [0.93–0.96]
	D	0.93 [0.83–0.97]	0.82 [0.56–0.93]	0.84 [0.60–0.93]	0.43 [0–0.77]	0.88 [0.70–0.95]	0.84 [0.39–0.94]	0.63 [0.12–0.85]	0.88 [0.83–0.91]
	*f*	0.94 [0.85–0.98]	0.86 [0.65–0.94]	0.90 [0.71–0.96]	0.78 [0.46–0.91]	0.66 [0.18–0.86]	0.83 [0–0.95]	0.81 [0.54–0.92]	0.85 [0.78–0.89]
	D*	0.90 [0.77–0.96]	0.71 [0.31–0.88]	0.84 [0.60–0.93]	0.66 [0.18–0.86]	0.61 [0.01–0.84]	0.62 [0–0.87]	0.66 [0.18–0.86]	0.76 [0.67–0.83]
BA-LA	ADC	[-0.30–0.29]	[-0.48–0.38]	[-0.56–0.44]	[-0.45–0.27]	[-0.39–0.39]	[-0.25–0.48]	[-0.15–0.21]	[-0.39–0.38]
	D	[-0.26–0.27]	[-0.44–0.39]	[-0.32–0.23]	[-0.61–0.48]	[-0.30–0.28]	[-0.23–0.10]	[-0.07–0.11]	[-0.35–0.30]
	*f*	[-0.06–0.07]	[-0.12–0.12]	[-0.06–0.10]	[-0.14–0.17]	[-0.12–0.17]	[-0.03–0.12]	[-0.08–0.09]	[-0.09–0.13]
	D*	[-10.5–8.6]	[-22.9–16.5]	[-12.3–12.1]	[-8.8–13.3]	[-7.2–13.8]	[-5.8–16.7]	[-17.2–13.2]	[-13.4–14.8]
Bias (mean) [95% CI]	ADC	0 [-0.07–0.07]	-0.05 [-0.15–0.05]	-0.06 [-0.18–0.06]	-0.09 [-0.18–-0.01]	0 [-0.09–0.09]	0.11 [0.03–0.20]	0.03 [-0.01–0.07]	-0.01 [-0.04–0.02]
	D	0.01 [-0.05–0.07]	-0.02 [-0.12–0.07]	-0.05 [-0.11–0.02]	-0.06 [-0.19–0.06]	-0.01 [-0.08–0.06]	-0.06 [-0.1–-0.03]	0.02 [0.00–0.04]	-0.03 [-0.05–0.00]
	*f*	0 [-0.01–0.02]	0 [-0.03–0.03]	0.02 [0–0.04]	0.02 [-0.02–0.05]	0.02 [-0.01–0.05]	0.05 [0.03–0.06]	0.01 [-0.01–0.03]	0.02 [0.01–0.03]
	D*	-0.98 [-3.20–1.24]	-3.20 [-7.78–1.38]	-0.10 [-2.94–2.74]	2.28 [-0.29–4.84]	3.31 [0.87–5.76]	5.43 [2.82–8.04]	-1.96 [-5.49–1.56]	0.68 [-0.49–1.85]

*CI* confidence interval, *ADC* apparent diffusion coefficient, *D* ‘true’ diffusion coefficient, D* pseudodiffusion coefficient, *f* perfusion fraction, *ROI* region of interest

## Discussion

Our study allowed evaluation of the robustness of quantitative assessment of IVIM parameters in the orbit. Test-retest repeatability of IVIM in the orbital tumours was good for ADC and D, but was poor for *f* and especially D*. Inter-observer repeatability agreement was almost perfect for all IVIM values in orbital tumours.

Our study was compliant with the Quantitative Imaging Biomarkers Alliance (QIBA) guidelines [25–27] to best evaluate technical performance of IVIM in orbital imaging. IVIM is a relatively new imaging technique and seems promising in the characterisation and evaluation of tumours, but proportionally few studies have evaluated its accuracy, repeatability or reproducibility. Therefore, we decided to perform a study to quantify the variability and possible source of error related to the technique in the orbit before further evaluation of its clinical relevance or impact in clinical practice [26, 27].

DWI and ADC repeatability have already been evaluated over time in head and neck or brain imaging [21, 28], but only few studies have evaluated the IVIM repeatability in these domains [9, 10] and none in the orbit to the best of our knowledge. A large majority of previously published studies evaluating the IVIM accuracy, repeatability or reproducibility were performed in the abdomen, especially in the liver. They showed a higher repeatability for ADC and D parameters than for perfusion-related *f* and D* parameters. The pseudo-diffusion coefficient D* appeared to be the least reproducible parameter among IVIM metrics, with the coefficient of variation ranging from 24.8% to 193.8% [11–14, 29], whereas the true diffusion coefficient D appeared to be the most reproducible parameter in the liver and in the kidney [14, 30, 31]. Although a recent multicentre study showed significant discrepancies of IVIM values between different MR imagers across multiple centres [15], studies evaluating the use of this technique in the abdomen concluded that IVIM parameters could be clinically relevant [14, 30, 31]. Our results are in agreement with these studies, with an acceptable test-retest repeatability for D in both orbital and extra-orbital structures (mean 22%, range 14–29%) similar to ADC (mean 22%, range 12–33%), and a poor repeatability for *f* and D* (mean 57%, range 43–75% and mean 130%, range 110–160%) [14, 30, 31]. A few hypotheses may explain these results. First, the IVIM model results from an oversimplification as it does not consider the exchanges between intra and extra-vascular compartments nor the possibility of a diffusion anisotropy and it does not distinguish arterial from venous flows, which may impair the accurate estimation of the two parameters related to blood flow, *f* and D* [2, 32]. Second, blood water comprises less than 5% of the total tissue water in the orbit as in many organs, therefore most of the IVIM signal arises from extravascular water and hence is theoretically uninformative in assessing blood flow by the IVIM method. The dynamic range for the measurement of *f* and D* is poor and the quality of parameter estimates depends upon a number of factors including the number of b values obtained, especially of low b values, and the signal-to-noise ratio, which has to be high enough, therefore requiring a high-field MRI [32]. Third, there is no direct measurement of the IVIM parameters but different methods to calculate them from exponential decay data, such as the nonlinear least squares or the Bayesian probability theory. Although reported to be more accurate, the Bayesian approach provides only a representation of the uncertainty in the parameters estimates in the form of a probability density function [33].

ADC and D fulfil the QIBA quality criteria [34]. In the literature, a 20% within-subject coefficient of variation is considered as a good threshold to use a biomarker in a clinical practice, because it suggests that a change of approximately 40% is required in a single subject to be considered that this difference may be related to a biological mechanism and not to the intrinsic variability of the technique [26].

Some technical challenges are common to abdominal and orbital imaging and affect the quality of IVIM, such as the presence of motion and susceptibility artefacts [13, 15, 30, 35]. The use of a high-field MRI is preferable in orbital and ocular imaging because of its higher signal-to-noise ratio and contrast-to-noise ratio as well as its better resolution, fully adapted for a small structure like the eye [35, 36]. However, large soft-tissue and air interfaces resulting in susceptibility effects are more pronounced at 3T and can provoke image distortions, leading to a loss of image quality [35]. In order to minimize motion artefacts in the orbit, we asked the patients to keep their eyes open during the exam and to stare at a fixed point. The use of a cued-blinking protocol including a regular break every 3 s, in which acquisition is automatically paused and the subject is instructed to blink, might be another strategy to reduce motion artefacts [37]. We also used parallel imaging techniques to decrease these artefacts, and bipolar diffusion gradients to attenuate eddy currents intrinsic to the process. Good visual quality of the sequence was determined in all of our patients by the two readers, and the estimated SNR at b= 2,000 s/mm^2^ was good, almost twice the minimal SNR recommended for reliable IVIM imaging [10]. No patient had to be secondarily excluded from the study because of artefacts masking or distorting orbital tumours.

Regarding the optimal type of ROI for analysing an orbital mass, the test-retest repeatability and the inter-observer agreements were slightly better with the use of a large freehand ROI encompassing the largest area inside the lesion versus a small circular ROI, especially for the D* parameter. The careful delineation of the entire lesion appeared to be more robust for analysis. These results are in agreement with other studies which evaluated the influence of the type, size, and position of tumour ROI on perfusion values, with a greater reliability and repeatability of large ROIs outlining entire tumours [38].

We investigated which intra-orbital or extra-orbital structures might be a good structure of reference for the quantitative use of IVIM values when diagnosing orbital diseases, if normalization of parameters between subjects was needed, such as it has been proposed in gynaecological tumours (using the outer myometrium) [39] or brain tumours (using a contralateral healthy area) [40]. The repeatability of the ADC and D parameters in the temporal muscle was excellent, with a low CV and a high ICC. This muscle is bigger than extra-ocular muscles or the lacrimal gland and is therefore easier to see. It is an immobile structure and it is less prone to image distortion compared to orbital structures. Moreover, it is almost always preserved, even in case of orbital diseases involving both eyes, thus being more reliable as a structure of reference.

In our study, D values were consistently lower than the ADC values despite a clear linear relation between these two values, strongly supporting the existence of both a diffusional component in the orbital tumours evaluated by the D value and a non-negligible perfusional component evaluated by *f* and D*. However, perfusion-related parameter changes will require caution in their interpretation in future studies considering their poor repeatabilities. On the other hand, ADC and D showed a good repeatability in our study, suggesting that these parameters might be more reliable and reproducible quantitative biomarkers to characterise orbital tumours, quantify the severity of the disease and predict its course, but also to evaluate therapeutic responses under treatments like chemotherapy for lymphomas or prototherapy for ocular melanomas.

Our study had some limitations. First, we included a relatively small number of patients recruited from a single centre, thus preventing us from performing analysis of the reproducibility of the IVIM technique on different MR devices or centres, which would be an important step before further large multicentre studies. Second, the time delay between the first and second IVIM acquisition was short, limiting the ability to evaluate all potential factors of variability and minimising the effects of other variance components. Third, we considered the orbit contralateral to the side of pathology as healthy, although most of the cases imaged were inflammatory diseases or lymphomas, which can affect both sides.

In conclusion, our study showed the feasibility and robustness of quantitative assessment of IVIM parameters in the orbit. Test-retest repeatability in orbital tumours was good for ADC and D, but was poor for *f* and especially for D*, whereas the inter-observer agreement repeatability was almost perfect for all values. Further research would be needed to improve MR acquisition and to increase the repeatability of perfusion-related IVIM parameters, and clinical studies must be performed to assess its potential utility in clinical practice.

## Electronic supplementary material

Below is the link to the electronic supplementary material.

ESM 1 (DOCX 19997 kb)

## Abbreviations

ADC: Apparent diffusion coefficient
BA-LA: Bland-Altman limits of agreements
CV: Coefficient of variation
D*: Pseudodiffusion coefficient
D: ‘True’ diffusion coefficient
DWI: Diffusion-weighted imaging
*f*: Perfusion fraction
ICC: Intra-class correlation coefficient
IVIM: Intravoxel incoherent motion
MRI: Magnetic resonance imaging
ROI: Region of interest
