# Supplementary material for: Repeatability of apparent diffusion coefficient and intravoxel incoherent motion parameters at 3.0 Tesla in orbital lesions
Source: Eur Radiol. 2017 Jul 4;27(12):5094–103. doi: 10.1007/s00330-017-4933-6 (PMC5674133; doi:10.1007/s00330-017-4933-6)
Supplement: Supplementary file 1 — (DOCX 19997 kb) [file 330_2017_4933_MOESM1_ESM.docx]

**
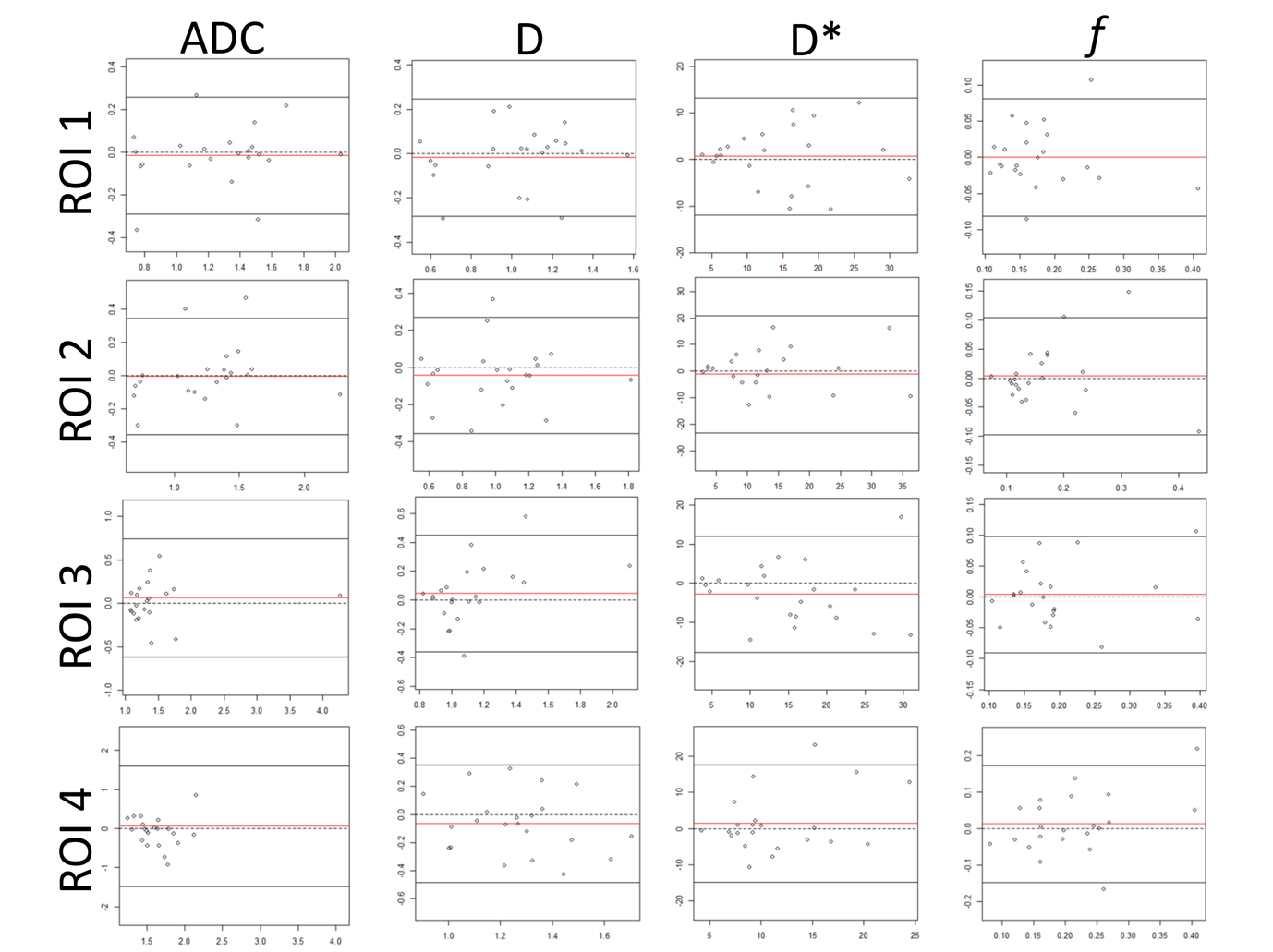
**

**
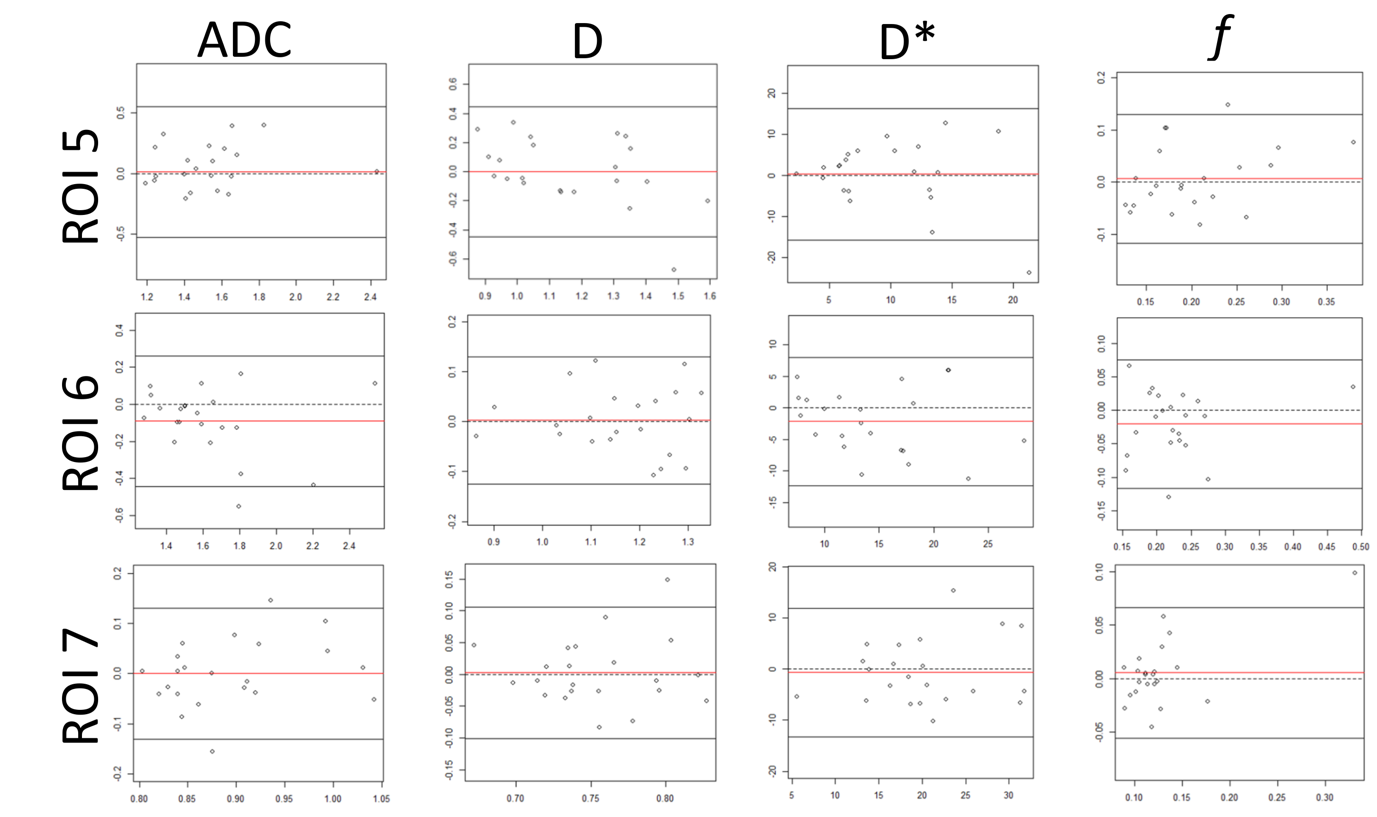
Supplementary Figure 1.** Bland-Altman plots showing test-retest repeatability of ADC, D, D*, *f* in the orbital mass (ROI 1 and 2) and in the lacrymal gland and extra-ocular muscles (ROI 3, 4, 5) and in the temporal muscle and the temporal lobe (ROI 6 and 7). Top and bottom lines correspond to 95% limits of agreement and central line to the mean difference. The X-axis represents the average of observations and the Y-axis the differences of observations.


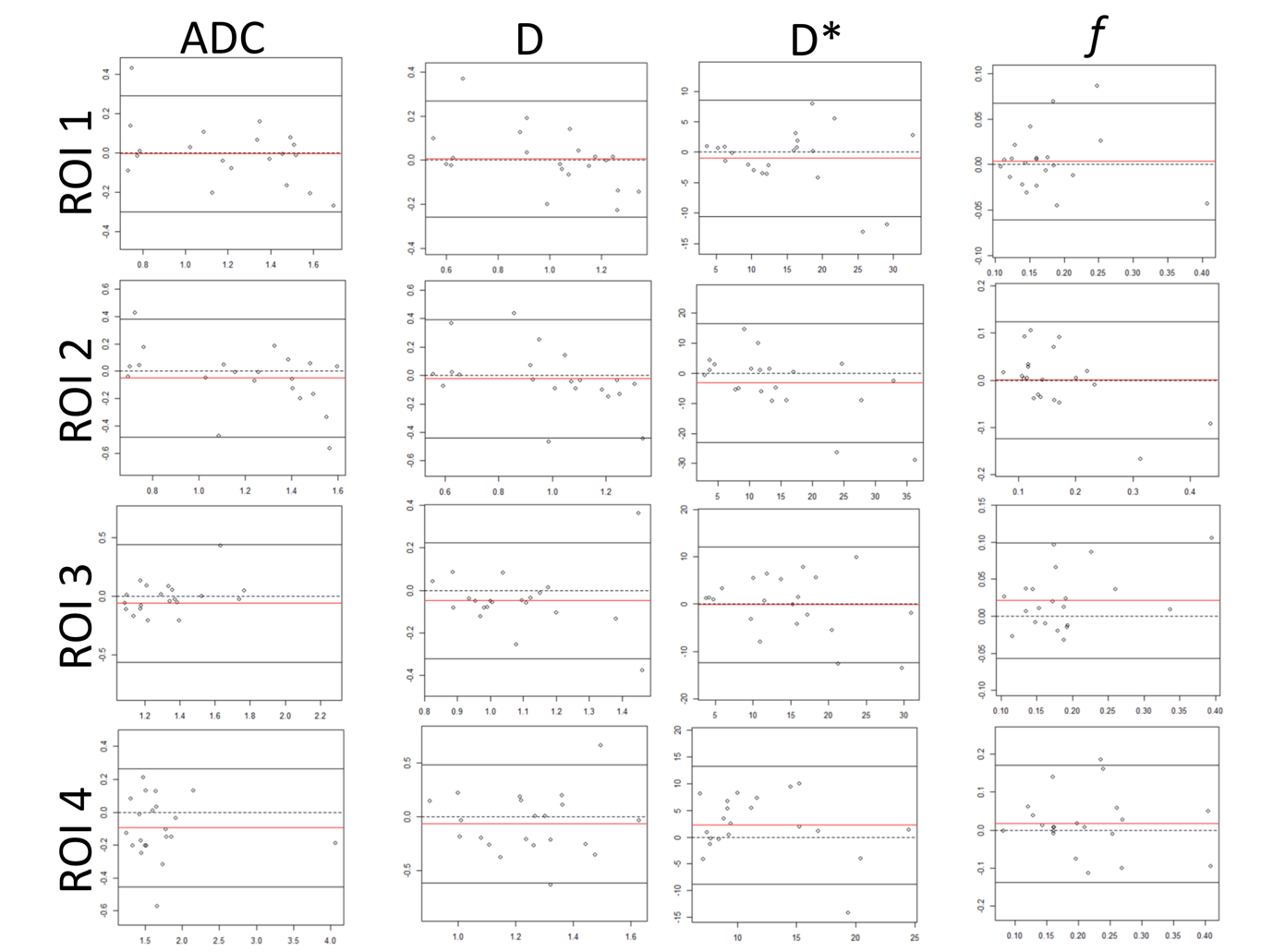


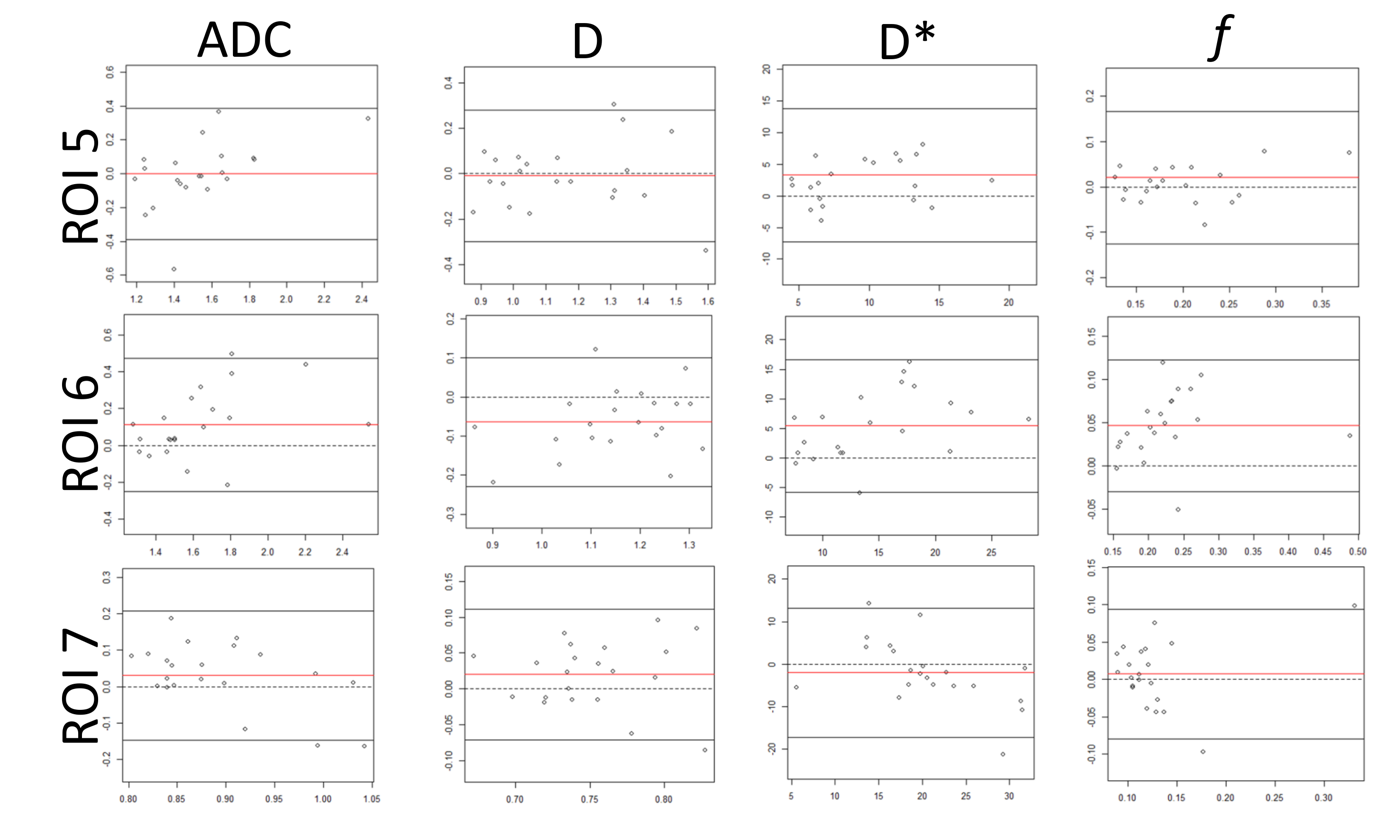


**Supplementary Figure 2.** Bland-Altman plots showing inter-observer repeatability agreement of ADC, D, D*, *f* in the orbital mass (ROI 1 and 2) and in the lacrymal gland and extra-ocular muscles (ROI 3, 4, 5) and in the temporal muscle and the temporal lobe (ROI 6 and 7). Top and bottom lines correspond to 95% limits of agreement and central line to the mean difference. The X-axis represents the average of observations and the Y-axis the differences of observations
